# In vivo visualization of PARP inhibitor pharmacodynamics

**DOI:** 10.1172/jci.insight.146592

**Published:** 2021-04-22

**Authors:** Elizabeth S. McDonald, Austin R. Pantel, Payal D. Shah, Michael D. Farwell, Amy S. Clark, Robert K. Doot, Daniel A. Pryma, Sean D. Carlin

**Affiliations:** 1 Division of Breast Imaging, Department of Radiology,; 2 Division of Nuclear Medicine Imaging and Therapy, Department of Radiology, and; 3 Division of Hematology/Oncology, Department of Medicine, University of Pennsylvania, Philadelphia, Pennsylvania, USA.

**Keywords:** Oncology, Diagnostic imaging

## Abstract

**BACKGROUND:**

[^18^F]FluorThanatrace ([^18^F]FTT) is a radiolabeled poly (adenosine diphosphate-ribose) polymerase inhibitor (PARPi) that enables noninvasive quantification of PARP with potential to serve as a biomarker for patient selection for PARPi therapy. Here we report for the first time to our knowledge noninvasive in vivo visualization of drug-target engagement during PARPi treatment.

**METHODS:**

Two single-arm, prospective, nonrandomized clinical trials were conducted at the University of Pennsylvania from May 2017 to March 2020. PARP expression in breast cancer was assessed in vivo via [^18^F]FTT PET before and after initiation of PARPi treatment and in vitro via [^125^I]KX1 (an analog of [^18^F]FTT) binding to surgically removed breast cancer.

**RESULTS:**

Thirteen patients had baseline [^18^F]FTT PET. Nine of these then had resection and in vitro evaluation of [^18^F]FTT uptake with an analog and uptake was blocked with PARPi. Of the other 4 patients, 3 had [^18^F]FTT PET uptake, and all had uptake blocked with treatment with a therapeutic PARPi. Initial in vivo [^18^F]FTT tumor uptake ranged from undetectable to robust. Following initiation of PARPi therapy, [^18^F]FTT uptake was not detectable above background in all cases. In vitro tumor treatment with a PARPi resulted in 82% reduction in [^125^I]KX1 binding.

**CONCLUSION:**

[^18^F]FTT noninvasively quantifies PARP-1 expression. Early results indicate ability to visualize PARPi drug-target engagement in vivo and suggest the utility of further study to test [^18^F]FTT PET as a predictive and pharmacodynamic biomarker.

**TRIAL REGISTRATION:**

ClinicalTrials.gov identifiers NCT03083288 and NCT03846167.

**FUNDING:**

Metavivor Translational Research Award, Susan G. Komen for the Cure (CCR 16376362), Department of Defense BC190315, and Abramson Cancer Center Breakthrough Bike Challenge.

## Abstract 2

Human PARP inhibitor drug-target engagement can be visualized, with future possibilities including a precision diagnostic and drug discovery tool.

## Graphical abstract

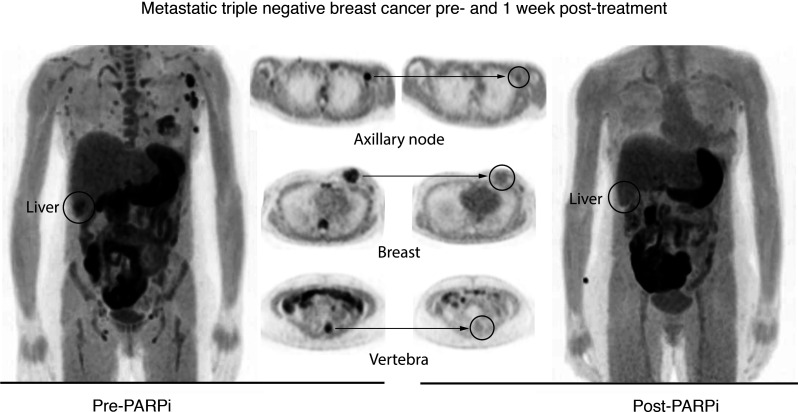

## Introduction

Poly (adenosine diphosphate-ribose) polymerase inhibitors (PARPi) show clinical efficacy in individuals with breast cancer and *BRCA1/2* mutations (1). Still, mutation status is not universally predictive of response, and some patients without germline mutations derive significant benefit (2, 3). Heterogeneous clinical response to PARPi, even within trials using mutation status and other homologous recombination deficiency biomarkers, creates a need for a predictive biomarker for PARPi therapy. [^18^F]FluorThanatrace ([^18^F]FTT) has a chemical structure similar to therapeutic PARPi and is radiolabeled with the positron emitter, ^18^F, for PET imaging (4, 5). Human dosimetry of [^18^F]FTT in 8 cancer patients was previously reported by Michel et al. to be comparable to other radiotracers currently in clinical use, with a table of estimated [^18^F]FTT residence times in organs (Supplemental Table 3 in ref. 5). [^125^I]KX1 is an analog with a longer half-life used for in vitro radioligand binding assays (6). Preclinical studies show high affinity and specificity of these tracers for PARP-1 (6, 7). In vitro data demonstrate that the level of PARP-1 correlates positively with cytotoxicity of PARPi and that PARP-1 expression is required for PARPi efficacy (6–9). We previously reported on imaging and quantifying PARP using [^18^F]FTT in breast and ovarian cancer (7, 10). This study builds on that body of work by demonstrating for the first time to our knowledge in vivo visualization of PARPi binding and subsequent PARP-1 suppression, with underlying biologic heterogeneity between tumors of similar molecular subtypes.

## Results

**Figure 1:**
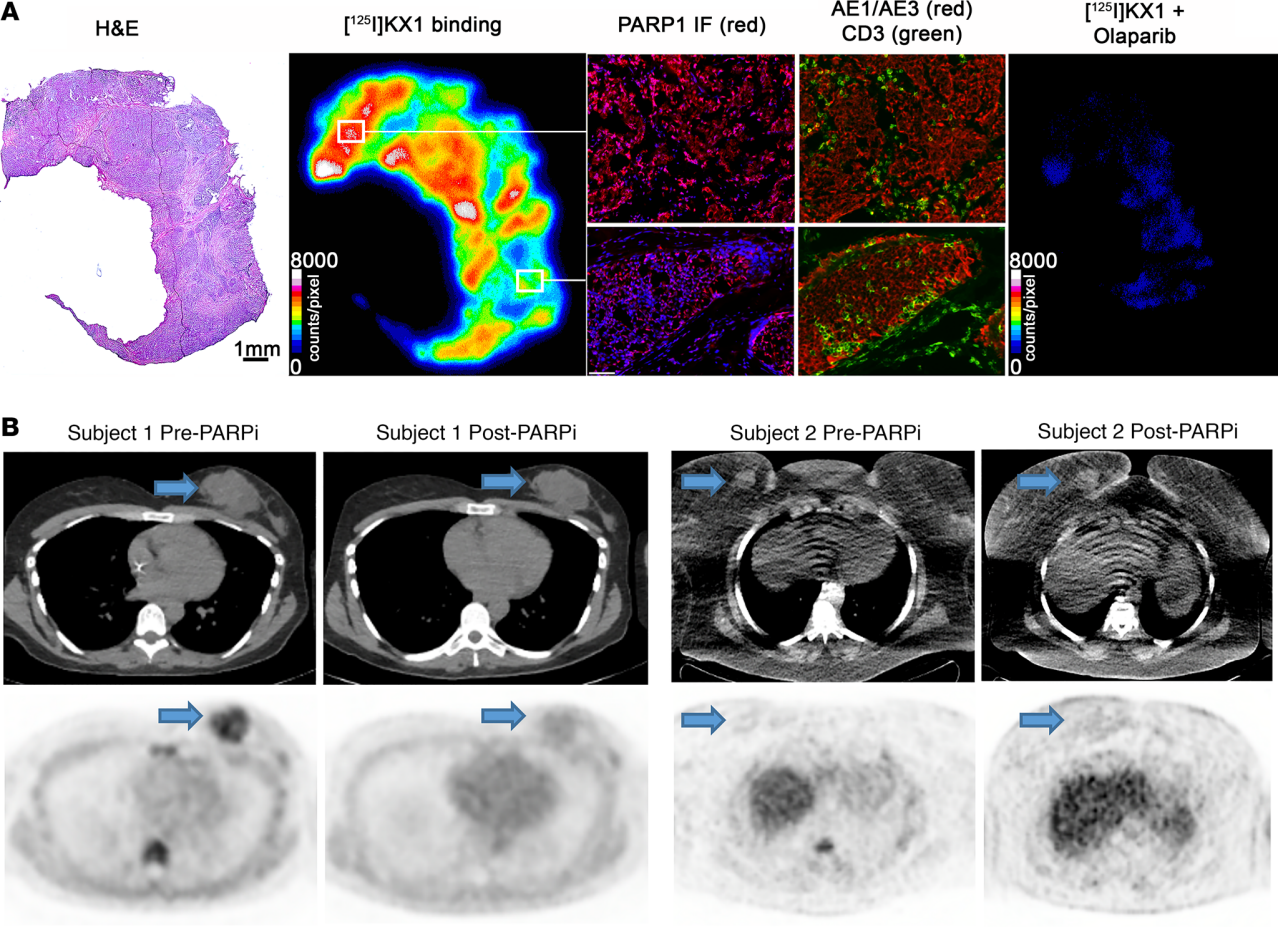
Baseline expression and subsequent suppression of PARP-1 after PARPi treatment in breast cancer. (**A**) Representative whole-tumor section demonstrates suppression of PARP radiotracer uptake with olaparib. Quantitative radioligand binding analysis (autoradiography) was followed by hematoxylin and eosin (H&E) staining. Contiguous cryosections were used to perform chromogenic PARP-1 immunofluorescence (red) with DAPI counterstain (blue). AE1/AE3 staining was performed to discriminate epithelial tumor cells (red), and CD3 staining was used to identify tumor-infiltrating T cells (green). Autoradiography demonstrates heterogeneity of PARP-1 expression at the microscopic level with spatial concordance between the intensity of [^125^I]KX1 uptake and expression of PARP-1 measured by immunofluorescence. [^125^I]KX1 plus 20 µM olaparib on a sequential section demonstrates tracer reduction to background levels. Scale bar on whole specimen H&E-stained slide is 1 mm. (**B**) [^18^F]FTT PET/CT image taken before and approximately 1 week after PARPi treatment for 2 women with advanced triple-negative breast cancer. Subject 1 had moderate [^18^F]FTT uptake pretherapy (SUV_max_ breast 4.7 g/mL) and blockade of uptake posttherapy (SUV_max_ breast 2.4 g/mL) and went on to have response to PARPi. Subject 2 had minimal uptake pretherapy (SUV_max_ breast 2.3 g/mL) and similar uptake posttherapy (SUV_max_ breast 2.4 g/mL) and had progression on PARPi.

An ex vivo PARPi competition assay was performed in tumors from 9 subjects with correlative in vivo PARP-1 imaging. There was an average of 82% (median 88%, range 55%–99%) suppression of [^125^I]KX1 uptake when olaparib was added to available frozen tumor tissue as shown for a representative subject. Spatial concordance of PARP-1 intratumor heterogeneity and [^125^I]KX1 was also demonstrated (Figure 1A).

Four study participants with stage III/IV breast cancer planning to receive PARPi treatment consented and completed pre- and post-PARPi [^18^F]FTT PET/CT. Age range was 41 to 71 (median 52). The histology of the primary breast malignancy was invasive ductal carcinoma for all patients. There were 3 triple-negative tumors and 1 estrogen receptor–positive tumor. Baseline PET imaging in 3 patients demonstrated moderate [^18^F]FTT uptake at sites of disease (maximum standardized uptake value [SUV_max_] range 4.2–6.8), with subsequent stable disease or tumor regression after PARPi. One subject did not have [^18^F]FTT uptake above regional background in any tumor (Figure 1B), with subsequent enlargement of the breast tumor and eventual distant disease and death within 1 year. After PARPi initiation, tumor [^18^F]FTT uptake was at background levels for all patients, with representative postimages in Figure 1B and the graphical abstract. There was intrasubject heterogeneity of pretreatment uptake as demonstrated in the graphical abstract. In this study subject, pretreatment SUV_max_ were 4.7 (breast), 7.7 (axillary node), 4.7 (spine), and 5.4 g/mL (liver), and posttreatment SUV_max_ were 2.4 (breast), 2.2 (axillary node), 2.3 (spine), and 2.9 g/mL (liver).

## Discussion

In breast cancer, PARPi are an important treatment option for *BRCA*-associated human epidermal growth factor receptor 2–negative metastatic disease and may soon be used in the neoadjuvant setting. Response to PARPi, though, remains variable, and a reliable predictive biomarker represents an unmet clinical need. Additionally, new, more effective drugs targeting PARP expression are in development, and an in vivo tool to quantify novel pharmacodynamics could help facilitate early evaluation of efficacy.

The data presented here leverage prior work indicating that [^18^F]FTT PET can measure regional PARP expression (6, 7) and demonstrate that uptake in breast cancer decreased to background after introducing a pharmacologic PARPi, building on in vitro assays supporting the use of [^18^F]FTT PET as a measure of drug-target engagement. [^18^F]FTT uptake at baseline signifies drug-target expression; abrogation of signal after PARPi therapy represents drug-target engagement. Both of these measures could potentially serve as biomarkers for PARPi response, and preclinical work suggests PARP-1 is required for PARPi cytotoxicity (6–9). These results provide proof-of-concept rationale that target engagement during PARPi treatment can be visualized and suggest the potential usefulness of [18F]FTT PET as a response biomarker and tool to visualize pharmacodynamic effects of PARPi compounds. Future studies will investigate the predictive value of this tracer for PARP therapy and whether the observed heterogeneity of initial uptake or uptake suppression after treatment corresponds to mixed response at specific sites of disease.

## Methods

### Study design.

Study participants were recruited at the University of Pennsylvania for NCT03846167 July 2019 to March 2020. Inclusion criteria were biopsy-proved breast cancer, consent for tissue analysis, and willingness to undergo [^18^F]FTT PET/CT before and after PARPi treatment. Four potential subjects were approached for the pilot study, provided written informed consent, and participated in the trial. Nine additional participants, described previously (10), underwent a [^18^F]FTT PET/CT scan as part of NCT03083288 with untreated tumor collection for PARP radioligand analysis. Testing for *BRCA1/2* was performed on all subjects, with 1 *BRCA1* mutation identified (10).

### Tissue preparation.

Freshly excised surgical tissue was immediately embedded in optimal cutting temperature (Thermo Fisher Scientific) and flash-frozen in isopentane/liquid nitrogen. Tissues were stored at –80°C and contiguous 10 μm cryosections cut for analysis.

### Immunofluorescence staining.

The 10 μm sections of frozen tissue were stained with rabbit anti-human PARP (clone 46D11, Cell Signaling Technology 9532, 1:1000), with secondary detection using rabbit Alexa Fluor 568 (Life Technologies, Thermo Fisher Scientific, A11036, 1:250). DAPI (Thermo Fisher Scientific 62248, 0.5 μg/mL) was a nuclear counterstain. Contiguous 10 μm sections of frozen tissue were stained using mouse anti-human CD3 (clone LN10, Leica PA0122, undiluted) and mouse anti-human multi-cytokeratin (clones AE1 and AE3, Leica NCL-L-AE1/3, 1:400). CD3 visualization was done using Opal HRP Polymer (PerkinElmer ARH1001EA) and Opal 520 Reagent Pack (PerkinElmer FP1487001KT, 1:100). Detection of cytokeratin was done by incubating with goat anti-mouse Alexa Fluor 647 (Life Technologies, Thermo Fisher Scientific, A21236, 1:250). Leica Biosystems BOND-III (Leica Microsystems) was utilized, and heat-induced epitope retrieval with BOND Epitope Retrieval Solution 2 (Leica Microsystems AR9640) was done for 20 minutes. Slides were mounted in ProLong Gold (Life Technologies, Thermo Fisher Scientific, P36961). The primary monoclonal PARP-1 antibody was validated on fixed and frozen tissue as a strong nuclear signal across a variety of normal tissues known to express PARP-1 prior to use.

### Radioligand binding assay.

Contiguous 10 μm cryosections identical to those used for immunofluorescence were incubated with [^125^I]KX1 (50 nM), in the absence or presence of 20 μM olaparib (Selleckchem). After 60 minutes, sections were washed, dried, and assessed for bound radioligand by exposing to a storage phosphor plate (BAS-IP SR, Thermo Fisher Scientific). Quantitative autoradiographic images were acquired using GE Healthcare Typhoon FLA 7000 plate reader and analyzed using GE Healthcare ImageQuant 8.1 software. Sequential sections were stained with H&E and scanned as above.

### [^18^F]FTT PET/CT imaging and analysis.

[^18^F]FTT was synthesized at the University of Pennsylvania Cyclotron Facility as previously described (10). Study subjects were scanned on an Ingenuity TF PET/CT (Philips Healthcare). Quantitative analysis of PET images was done by a fellowship-trained nuclear medicine physician and fellowship-trained breast radiologist from a 20-minute scan beginning 60 minutes after injection using MIM v6.7 (MIM Software Inc.). There were no study-related adverse events in study participants. Tumor uptake of [^18^F]FTT was reported as SUV_max_ in units of g/mL as previously reported for this tracer (7, 10). SUV_max_ was recorded from a spherical region of interest placed over known tumors with reference to CT, [^18^F]fluorodeoxyglucose-PET, and prior breast imaging studies, if applicable. The target lesion was the primary breast tumor. If already surgically removed (*n* *=* 1), the metastatic lesion with highest pretherapy SUV_max_ was the target lesion for pre- and posttherapy measurements.

### Statistics.

No statistical analyses outside of calculation of average and median values were applied to the data set in this manuscript.

### Study approval.

Studies were approved by the University of Pennsylvania Institutional Review Board (numbers 826390 and 832165), and written informed consent was obtained from participants.

## Author contributions

ESM was responsible for designing research studies, conducting experiments, acquiring data, analyzing data, and writing the manuscript; ARP was responsible for conducting experiments, acquiring data, analyzing data, and writing the manuscript; PDS was responsible for acquiring data and manuscript review; DAP was responsible for acquiring data and manuscript review; ASC was responsible for acquiring data and manuscript review; RKD was responsible for analyzing data and writing the manuscript; MDF was responsible for acquiring data and manuscript review; and SDC was responsible for designing research studies, conducting experiments, acquiring data, analyzing data, providing reagents, and writing the manuscript.

## Supplementary Material

Trial reporting checklists

ICMJE disclosure forms

## Version 1 04/22/2021

Electronic publication
